# Differential effects of anesthetics and sex on supraventricular electrophysiology and atrial fibrillation substrate in rats

**DOI:** 10.1038/s41684-025-01532-5

**Published:** 2025-03-26

**Authors:** Michael Murninkas, Or Levi, Sigal Elyagon, Aviv Komissar, Neta Marom, Alon Naumchik, Noam Dalal, Gideon Gradwohl, Yoram Etzion

**Affiliations:** 1 Cardiac Arrhythmia Research Laboratory, Department of Physiology and Cell Biology, Faculty of Health Sciences, Ben-Gurion University of the Negev, Beer-Sheva, Israel; 2 Regenerative Medicine and Stem Cell Research Center, Ben-Gurion University of the Negev, Beer-Sheva, Israel; 3 Medical Engineering Unit, The Jerusalem College of Technology, Jerusalem, Israel

**Keywords:** Atrial fibrillation, Electrocardiography - EKG, Atrial fibrillation

## Abstract

Rodents are increasingly used in atrial electrophysiology research, yet such studies are often performed under anesthesia owing to technical challenges. Here we developed an implantable device for comprehensive atrial studies in ambulatory rats and investigated the effects of commonly used anesthetics on supraventricular electrophysiology and arrhythmic substrate, comparing them with the unanesthetized state (UAS). Adult rats were evaluated 4 weeks after implantation. Studies were conducted in the UAS under 2% isoflurane (ISO) and under 40 mg/kg pentobarbital (PEN). Pacing protocols determined various parameters, including sinoatrial node recovery time, atrioventricular node effective refractory period and atrial effective refractory period. Arrhythmic substrate was assessed after 20 triggering bursts per condition, and arrhythmic tendency was analyzed manually and through the complexity ratio, an unbiased measure recently developed by our group. PEN mildly increased heart rate in both sexes, while ISO did not affect heart rate but prolonged the corrected sinus node recovery time in males. PEN increased atrioventricular node effective refractory period in both sexes, while ISO affected males only. Both ISO and PEN prolonged atrial effective refractory period compared with UAS in both sexes. Arrhythmic measures were higher in males and were attenuated by ISO and, to a lesser extent, by PEN in males only. The dominant frequency of arrhythmic events was reduced by both anesthetics in both sexes. These findings demonstrate a significant impact of commonly used anesthetics on rat supraventricular electrophysiology, with sex-based differences, highlighting the importance of methodologies that enable cardiac electrophysiology studies in unanesthetized rodents.

## Abstract (web summary)

The authors used an implantable electrophysiological device to assess the effects of two commonly used anesthetics (isoflurane and pentobarbital) on supraventricular electrophysiology and arrhythmic substrate in male and female rats.

## Main

Atrial fibrillation (AF), the most common sustained cardiac arrhythmia, presents a formidable medical challenge with substantial complications and an increased risk of mortality^1–3^. The pathophysiology of AF is complex and progressive^4^, influenced by mechanisms that alter the electrical and structural properties of the atrial myocardium^5,6^. Aging, along with common conditions such as hypertension, diabetes mellitus, obesity and obstructive sleep apnea, converge and increase the ‘AF substrate’, that is, the susceptibility of the atrial tissue to the recurrence and persistence of arrhythmia^7,8^. Despite ongoing research, a comprehensive understanding of the mechanisms involved in AF substrate formation in various clinical conditions remains elusive. Reliable biological models are crucial for better understanding the underlying mechanisms and for effectively testing new therapeutic strategies^9,10^. Although novel in vitro atrial models are evolving^11,12^, many challenges remain, including accurate differentiation, multicellularity and realistic electrical and mechanical functions. Therefore, animal models remain indispensable.

Historically, AF research relied almost exclusively on large animals. However, recent years have witnessed a rapid increase in the use of rodents for AF research, largely due to the remarkable ability to increase the AF substrate in these small mammals using clinically relevant insults^13–18^. Nonetheless, technical challenges associated with the small and delicate atrial anatomy limit most electrophysiological studies and AF induction protocols to either ex vivo preparations or the invasive insertion of atrial-pacing electrodes under deep anesthesia^19,20^. Two of the most widely used anesthetics in rodent AF studies are isoflurane (ISO) and pentobarbital (PEN) (for example, refs. ^21–24^). Although these agents enable experimental procedures to be performed, they can alter hemodynamic and cardiac electrophysiology parameters^19,25–27^. Thus, it is also likely that they can markedly modulate the arrhythmogenic substrate of atrial tissue. However, the limited ability to compare the atrial electrophysiology and arrhythmogenic substrate of anesthetized rodents with these parameters in rodents in the unanesthetized state (UAS) has, so far, prevented direct evaluation of this issue. In addition, there may be important sex-dependent variations in the effects of anesthetic agents on the cardiac electrophysiology of rodents^28–30^. However, to the best of our knowledge, any sex-related effects of anesthetics on supraventricular electrophysiology and AF inducibility in rodents have yet to be defined. Importantly, in humans, men are up to two times more likely than women to develop AF for reasons that are not yet understood^31^. Recent in vivo data indicate that male mice exhibit increased AF substrate relative to females, which may be regulated by the presence of androgens^31^. However, the situation in rats is unclear in this regard, while the effects of anesthetics on sex-dependent differences are also unknown.

In our group, we recognized the lack of appropriate tools to perform long-term cardiac electrophysiology studies in rodents. Therefore, we previously developed an implant system designed to make repeated pacing and electrophysiological recordings in freely moving rats and mice^32–36^. More recently, we improved this system in rats by introducing a miniature atrial quadripolar electrode composed of medical-grade silicon and fully biocompatible metal components. This electrode enables simultaneous atrial pacing and recording, exhibiting stable capture thresholds and high-resolution atrial recordings over extended periods of at least 8 weeks^37^. Moreover, the high resolution of the recorded atrial signals enabled us to advance arrhythmia analysis and develop an unbiased computerized approach to clean the atrial signal from ventricular mixing. Thereafter, we could readily evaluate the signal power spectrum as well as the irregularity of arrhythmic events^38^. Notably, while the atrial electrode was primarily designed for long-term pacing and recording purposes, we have found that, when implanted for several weeks, it gradually reduces the atrial effective refractory period (AERP) and concomitantly increases the AF substrate in adult male rats^36,37^. The mechanisms leading to this phenomenon have yet to be elucidated but presumably reflect mechanical loading related to the electrode’s weight or resistance to contractions, leading to localized atrial remodeling and AERP dispersion^37^. Regardless of the exact mechanism, the arrhythmogenic substrate that develops in this model mimics some important aspects of AF-related remodeling and opens a window of opportunity to evaluate the effect of multiple manipulations on the atrial-selective AF substrate obtained.

**Fig. 1:**
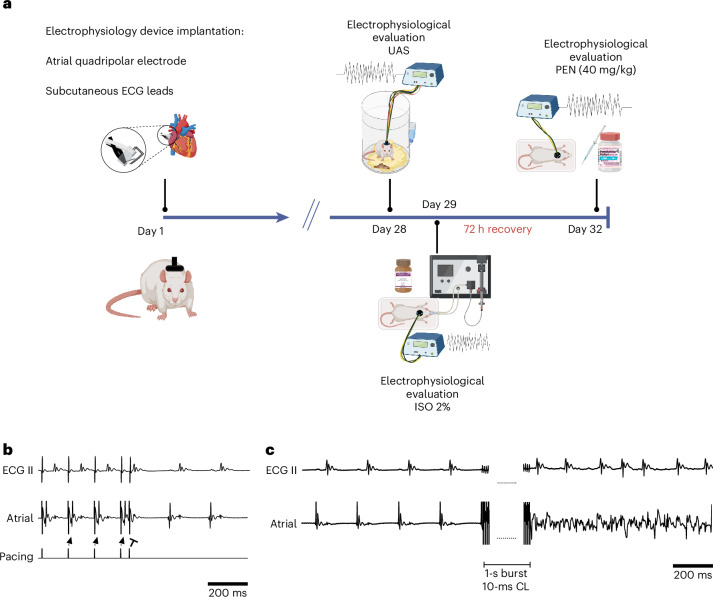
Experimental setup. **a**, Schematic representation of the experimental design. On day 1, each rat was implanted with an electrophysiology device comprising an atrial quadripolar device and three peripheral ECG leads, all connected to an eight-pin connector in the back of the rat. For additional details, see the Methods; please also see our previous publications for detailed descriptions and photographs^36,37^. **b**, A typical recording during the S1–S2 protocol, applied using the atrial quadripolar electrode. The arrowheads mark atrial capture, while the blunt arrow indicates failure of atrial capture during S2. **c**, A typical example of an AF episode induced by burst pacing using the atrial quadripolar electrode. Left, baseline ECG and atrial bipolar signals. Middle, burst pacing (1-s duration, 100 Hz, double threshold). Right, post-burst recording. Note the high resolution of the obtained atrial signals in **b** and **c**. Panel **a** was created with BioRender.com.

In the current study, taking advantage of our unique electrophysiology system capabilities, we explore the impact of various anesthetics on supraventricular electrophysiology and the differential impact of these agents on males versus females. For these purposes, adult rats of both sexes were evaluated 4 weeks after device implantation by repeated electrophysiological studies conducted under UAS, ISO (2%) and PEN (40 mg/kg) (Fig. 1). Our results indicate a notable impact of commonly used anesthetics on the supraventricular electrophysiology parameters and arrhythmogenic substrate of rats, which is often sex dependent. Overall, our findings provide valuable data on the complex effects of anesthetics and emphasize the importance of methodologies enabling rodent electrophysiology studies in the UAS.

## Results

### Supraventricular electrophysiology properties of unanesthetized rats differ markedly by sex

Four weeks after electrophysiology device implantation, we first analyzed the electrophysiological properties of male and female rats in the UAS. While we performed the implantation procedure on rats of similar weight, the males were notably heavier during the electrophysiology studies (Table 1), as expected. Basal electrocardiogram (ECG) recordings revealed a slower heart rate, as inferred from the longer mean RR interval, in males compared with females (182.20 ± 4.21 ms versus 168.60 ± 3.48 ms, respectively; *P* = 0.019, Student’s *t*-test). The PR interval was also slightly but significantly longer in males than in females (49.96 ± 1.15 ms versus 46.99 ± 0.90 ms; *P* = 0.049, Student’s *t*-test; Table 1).

**Table 1:** Differential effects of sex on the supraventricular electrophysiology and atrial arrhythmic susceptibility of unanesthetized rats

Parameter	Male (*n* = 13)	Female (*n* = 16)	*P* value
Animal weight (g)	386.5 ± 6.7	278.0 ± 5.3	<0.001***
RR interval (ms)	182.2 ± 4.21	168.6 ± 3.48	0.019*
PR interval (ms)	49.96 ± 1.15	46.99 ± 0.9	0.049*
CSNRT (ms)	20.29 ± 1.87	23.15 ± 1.69	0.267
AERP 70 CL (ms)	26.22 ± 1.45	31.56 ± 1.43	0.019*
AERP 100 CL (ms)	27.33 ± 1.48	31.10 ± 1.54	0.098
AERP 120 CL (ms)	26.11 ± 1.26	30.40 ± 1.59	0.053
AVERP 100 CL (ms)	75.08 ± 0.97	73.75 ± 1.67	0.534
AVERP 110 CL (ms)	73.31 ± 0.92	72.75 ± 1.78	0.797
AVERP 120 CL (ms)	73.38 ± 1.06	72.25 ± 1.73	0.602
AVERP 130 CL (ms)	71.92 ± 1.14	71.25 ± 1.57	0.742
AV 2:1 block (ms)	75.83 ± 1.72	70 ± 1.49	0.021*
AV Wenckebach block (ms)	90.83 ± 2.03	82.5 ± 1.34	0.004**
Regular SVT induction (%)	12.31 ± 2.69	10.63 ± 3.22	0.420
Regular SVT duration (s)	236.98 ± 140.37	125.55 ± 105.04	0.177
AF induction (%)	30.77 ± 6.72	9.69 ± 3.04	0.010*
AF duration (s)	48.57 ± 28.83	5.54 ± 1.89	0.016*
Mean CR	1.22 ± 0.03	1.1 ± 0.02	0.009**
Arrhythmic CR (%)	39.34 ± 5.83	16.81 ± 4.30	0.009**

Comparison between the supraventricular electrophysiological parameters of male and female rats obtained in the UAS 4 weeks after right-atrial quadripolar electrode implantation. Note the reduced AERP and markedly increased AF susceptibility in males compared with females. Mean CR refers to the average CR of the first 5 s post-induction bursts. The results of 20 induction bursts were averaged for each animal. Arrhythmic CR (%) refers to the percentage of post-burst windows with a CR greater than the arrhythmic threshold (1.236). For additional details, see the Methods. Values are given as the mean ± s.e.m. Statistical analysis: for the electrophysiological parameters, the Student’s *t*-test was applied; for arrhythmic parameters, the Mann–Whitney test was applied. *P < 0.05, **P < 0.01, ***P < 0.001.

In contrast with the RR interval, the corrected sinus node recovery time (CSNRT) obtained during programmed stimulation did not differ between males and females, suggesting similar sinoatrial (SA) nodal properties. The AERP measurements of both sexes revealed that there was an absence of typical rate adaptation over the entire range of basic cycle lengths (CLs) tested (120–70 ms). This was similar to our earlier finding in unanesthetized males^37^. However, for all basic CLs, the AERPs of males tended to be shorter relative to those of females, and this difference became significant once a basic CL of 70 ms was applied (26.2 ± 1.4 ms versus 31.5 ± 1.4 ms, respectively; *P* = 0.019). Regarding atrioventricular (AV) nodal function, AV node effective refractory period (AVERP) measurements revealed no significant differences between males and females over the entire range of tested basic CLs (130 to 100 ms). However, dynamic AV nodal properties (AV Wenckebach and AV 2:1 block) were significantly longer in males compared with females (*P* = 0.004 and *P* = 0.021, respectively, Student’s *t*-test; Table 1). Overall, our data demonstrate that, in the UAS, there are marked differences in heart rate, atrial AERP and dynamic AV nodal conduction properties between male and female rats.

### Unanesthetized males demonstrate markedly increased AF substrate

**Extended Data Fig. 1:**
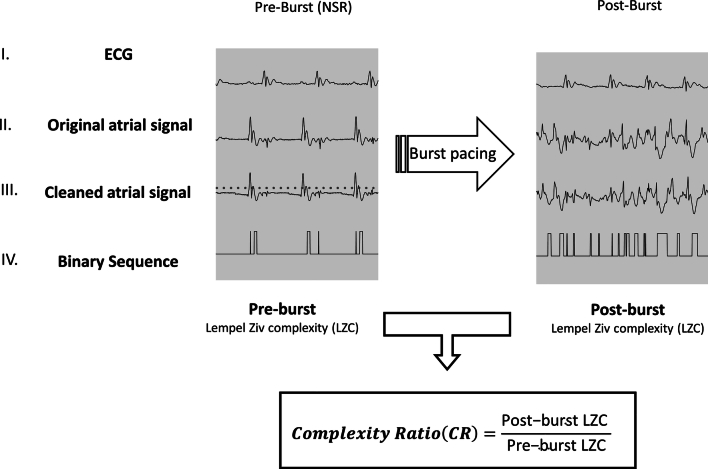
Complexity analysis of the rat atrial signal following ventricular signal subtraction. An example of a recording before (left) and after (right) a burst pacing protocol leading to an arrhythmic event. I: peripheral ECG, II: original atrial recording. III: Cleaned atrial recording following ventricular signal subtraction based on QRS detection in the ECG^38^. Dashed line represents the complexity analysis threshold. IV: binary sequence derived from the crossing of the amplitude threshold by the pure atrial signal. Post-burst binary sequence was analyzed in 1-s windows using the Lempel-Ziv complexity (LZC) algorithm^38^ (see also METHODS for details). The final complexity ratio (CR) for each window was calculated as the post-burst LZC of this window divided by the pre-burst LZC. As we have previously shown, CR > 1.236 could detect irregular arrhythmic signals in a highly efficient manner (AUC = 96.3%, Sensitivity=94.5%, Specificity=93.1%)^38^.

The application of burst pacing to induce arrhythmia revealed no sex-based differences in the induction or duration of regular supraventricular tachycardias (SVTs) (Table 1). However, we found markedly increased AF substrate in males compared with females. This result was noted when AF substrate parameters (induction and duration) were measured manually (AF induction: 30.77% ± 6.72% versus 9.69% ± 3.04% in males versus females, respectively, *P* = 0.010, Mann–Whitney test; AF duration: 48.57 ± 28.83 s versus 5.54 ± 1.89 s in males versus females, respectively, *P* = 0.016, Mann–Whitney test). In addition, we also applied our recently developed tool^38^ for the analysis of irregular arrhythmia based on complexity ratio (CR) measurements. In brief, the CR is an automated tool that detects irregularity of the atrial signals based on the utility of the Lempel–Ziv complexity algorithm, calculated in the pre- and post-burst pacing signals and expressed as a ratio between the post-burst and pre-burst values (Extended Data Fig. 1). The CR gets higher with increased irregularity and allows precise discrimination between irregular atrial arrhythmias (AF) and regular atrial rhythms. Indeed, the CR analysis in both sexes confirmed the manual analysis (mean CR: 1.22 ± 0.03 versus 1.10 ± 0.02 in males versus females, respectively, *P* = 0.009, Mann–Whitney test; arrhythmic CR seconds above the cutoff for AF: 39.34% ± 5.83% versus 16.81% ± 4.30% in males versus females, respectively, *P* = 0.009, Mann–Whitney test). Overall, the AF substrate related to our implanted device was found to be markedly increased in unanesthetized males compared with unanesthetized females (Discussion).

### Anesthetics markedly modulate the supraventricular electrophysiological properties of rats in a sex-dependent manner

**Fig. 2:**
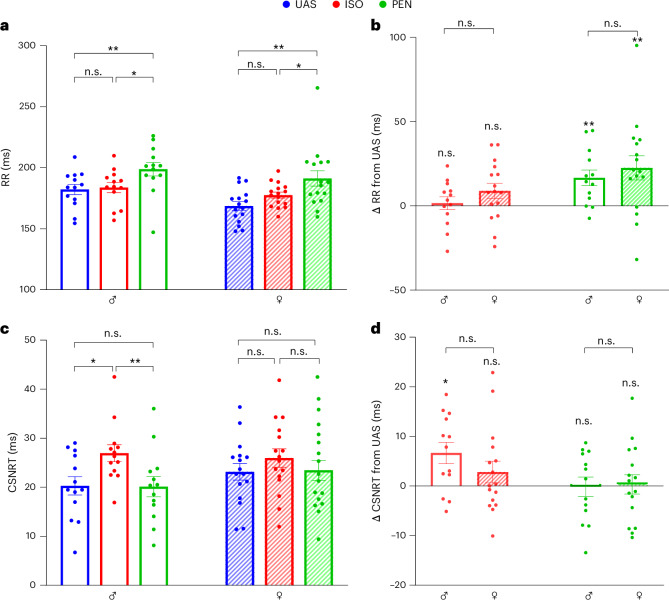
Heart rate and SA node properties are differentially affected by ISO and PEN in male and female rats. **a**, Comparison of RR intervals under UAS, ISO and PEN, stratified by sex. Note prolongation of RR intervals by PEN relative to UAS and ISO in both males and females. **b**, Comparison between males and females of Δ change in the RR interval relative to UAS under ISO and PEN conditions. **c**, Comparison of CSNRT under UAS, ISO and PEN, stratified by sex. Note that, in males only, there was marked prolongation of CSNRT under ISO relative to both UAS and PEN. **d**, Comparison between males and females of Δ change in the CSNRT relative to UAS under ISO and PEN conditions. Statistical analysis: normality was confirmed in **a** and **c**; thus, one-way ANOVA for repeated measurements was applied, followed by post-hoc Tukey’s multiple-comparison test. Normality was confirmed in **b** and **d**; thus, an unpaired Student’s *t*-test was applied to compare males and females. In all panels, *n* = 13 for males and *n* = 16 for females. All data are expressed as mean ± s.e.m. *P < 0.05, **P < 0.01. n.s., not significant.

**Fig. 3:**
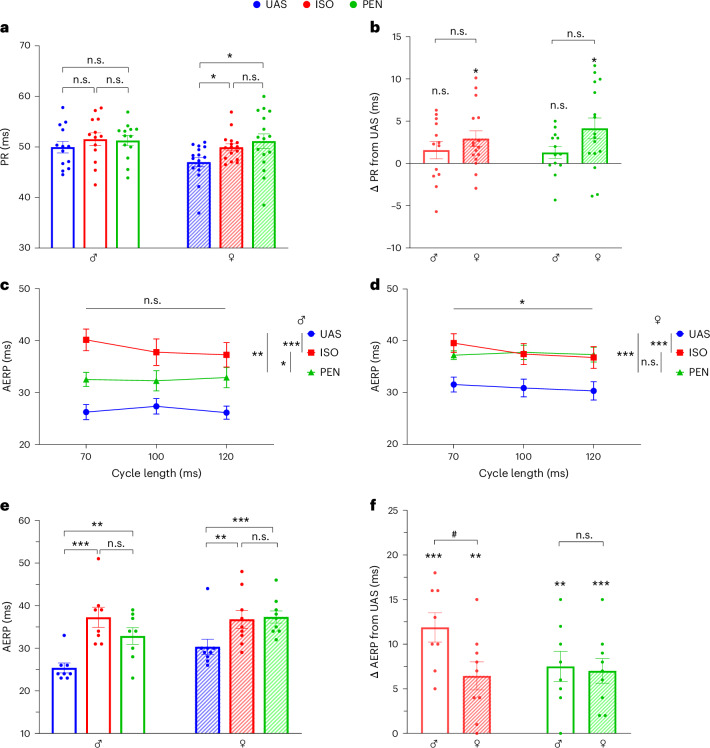
Anesthetics prolong the PR interval in females only and AERP in both sexes. **a**, Comparison of PR intervals under UAS, ISO and PEN, stratified by sex. Note the small but significant prolongation of the PR interval under ISO and PEN relative to UAS in females only. **b**, Comparison between males and females of Δ change in the PR interval relative to UAS under ISO and PEN conditions. The results were nonsignificant in this analysis. **c**,**d**, AERP as a function of basic CL in males (**c**) and females (**d**). Note the absence of rate adaptation in males and the small but significant reverse rate adaptation in females (that is, prolongation of AERP as the CL decreases). **e**, Comparison of AERP at 120 ms basic CL under UAS, ISO and PEN, stratified by sex. Note the prolongation of AERP by both ISO and PEN relative to UAS in both males and females. **f**, Comparison between males and females of Δ change in the AERP at 120 ms basic CL relative to UAS under ISO and PEN conditions. The Δ change in AERP was significant only for ISO. Statistical analysis: Normality was confirmed in **a** and **e**; thus, one-way ANOVA for repeated measurements was applied, followed by post-hoc Tukey’s multiple-comparison test. Normality was confirmed in **c** and **d**; thus, two-way ANOVA for repeated measurements was applied, followed by post-hoc Tukey’s multiple-comparison test. Normality was confirmed in **b**; thus, an unpaired Student’s *t*-test was applied to compare males and females. Normality was not confirmed in **f**; thus, the Mann–Whitney test was applied to compare males and females. In **a** and **b**, *n* = 13 for males and *n* = 16 for females. In **c**–**f**, *n* = 8 for males and *n* = 9 for females. All data are expressed as mean ± s.e.m. *P < 0.05, **P < 0.01, ***P < 0.001; ^#^*P* < 0.05 for Δ change between males and females.

Initially, we evaluated the effects of ISO and PEN on both male and female rats’ heart rate and SA nodal function (Fig. 2). There was no difference between the RR interval in ISO-treated or UAS rats, in either sex. By contrast, a prolonged RR interval was observed in PEN-treated rats compared with ISO-treated and UAS rats, in both sexes (Fig. 2a). A similar prolonging effect on the RR interval by PEN in both males and females was also seen when Δ changes from the UAS were compared for both sexes (Fig. 2b). Interestingly, although ISO did not affect the RR interval, it significantly prolonged the CSNRT in males compared with UAS and PEN-treated males (*P* = 0.02, *P* = 0.04, respectively, one-way analysis of variance (ANOVA) with Tukey’s multiple comparisons; Fig. 2c). Neither anesthetic had any effect on the CSNRT of females (Fig. 2c). Analysis comparing the Δ changes of CSNRT relative to UAS in both sexes did not reveal statistical significance (Fig. 2d). Anesthetics also modified the PR interval in a complex manner. While this parameter was insensitive to either anesthetic in males, the inherently shorter PR interval of females in the UAS (Table 1) was significantly prolonged by both anesthetics (*P* = 0.02, *P* = 0.01, respectively, one-way ANOVA with Tukey’s multiple comparisons test; Fig. 3a). However, comparison of the Δ changes of PR relative to UAS between males and females did not reach significance (Fig. 3b). Overall, the above findings indicate complex and differential effects of ISO and PEN that are also somewhat variable between males and females concerning CSNRT and the PR interval.

**Extended Data Fig. 2:**
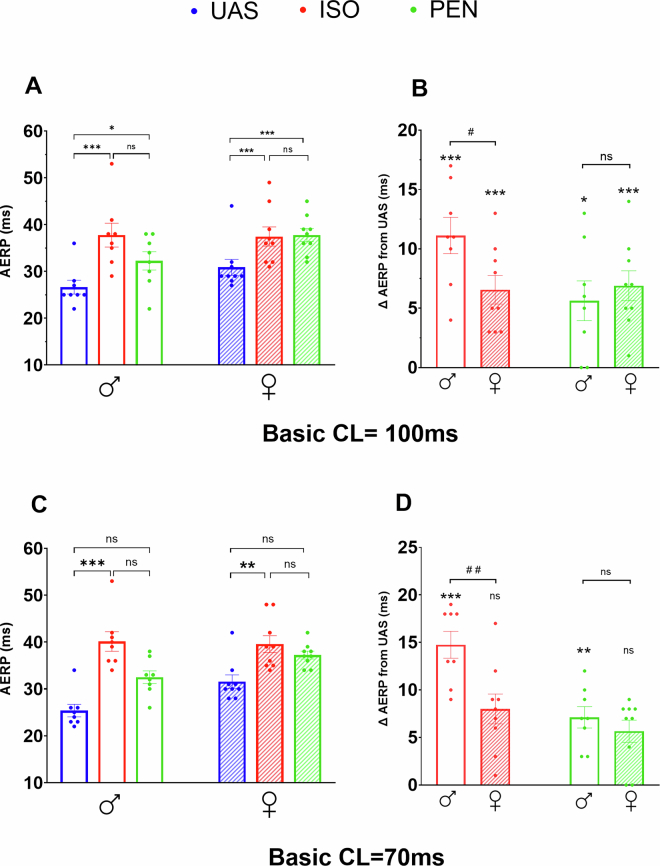
AERP analysis for various basic CLs. **A**: Comparison of AERP obtained with 100-ms basic CL under UAS, ISO and PEN, stratified by sex. **B**: Comparison between males and females. The Δ change in AERP obtained with 100-ms basic CL relative to UAS under ISO and PEN conditions. **C-D:** Similar representations as in **A-B** but for 70-ms basic CL. Similar data for 120-ms basic CL are presented in Fig. 3E, F in the main paper.

AERP was tested using three different basic CLs (70, 100 and 120 ms). Consistent with our previously reported findings^32,37^, we did not observe, under any of the conditions in either sex, indications of typical rate adaptation (that is, reduced AERP when the basic CL was decreased). However, while two-way ANOVA indicated a lack of any rate adaptation in males (Fig. 3c), a small but significant reverse rate adaptation (that is, increased AERP when the basic CL was decreased) was noted in females (Fig. 3d). Notably, both ISO and PEN markedly prolonged AERP in both sexes and for all the tested CLs (Fig. 3c,d). However, while the AERP-prolonging effect of ISO was increased relative to PEN in the males, both anesthetics had a similar prolonging effect in the females. Detailed analyses of the AERP findings for each CL are also shown for 120 ms CL and the other CLs (Fig. 3e and Extended Data Fig. 2, respectively), providing further support for the abovementioned findings. Comparison of the Δ changes of AERP relative to UAS also indicated increased effect of ISO in males relative to the females (Fig. 3f).

**Fig. 4:**
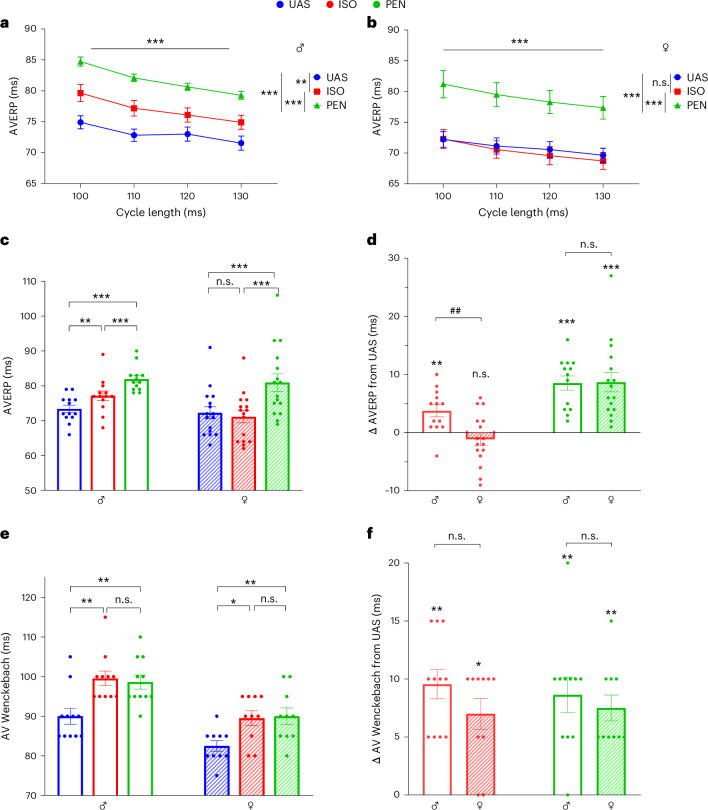
AV nodal properties are differentially affected by ISO and PEN in both sexes. **a**,**b**, AVERP as a function of basic CL in males (**a**) and females (**b**). Note the prolongation of AVERP as CL is decreased in both sexes and under all experimental conditions. Also, note the marked prolongation of AVERP by both ISO and PEN in males and by PEN only in females. **c**, Comparison of AVERP at 120 ms basic CL under UAS, ISO and PEN, stratified by sex. Note the prolongation of AVERP by both ISO and PEN relative to UAS in males and by PEN only in females. **d**, Comparison between males and females of the Δ change in AVERP relative to UAS under ISO and PEN conditions. Note the differing responses of males and females to ISO. **e**, Comparison of AV Wenckebach under UAS, ISO and PEN, stratified by sex. Note the prolongation of this dynamic parameter in both males and females relative to UAS. **f**, Comparison between males and females of the Δ change in AV Wenckebach relative to UAS under ISO and PEN conditions. Statistical analysis: normality was confirmed in **a** and **b**; thus, two-way ANOVA for repeated measurements was applied, followed by post-hoc Tukey’s multiple-comparison test. Normality was confirmed in **c**; thus, one-way ANOVA for repeated measurements was applied, followed by post-hoc Tukey’s multiple-comparison test. Normality was not confirmed in **e**; thus, Friedman’s test was applied, followed by Dunn’s multiple-comparison correction. Normality was not confirmed in **d** and **f**; thus, the Mann–Whitney test was applied to compare males and females. In **a**–**d**, *n* = 13 for males and *n* = 16 for females. In **e** and **f**, *n* = 11 for males and *n* = 10 for females. All data are expressed as mean ± s.e.m. *P < 0.05, **P < 0.01, ***P < 0.001; ^##^*P* < 0.01 for Δ change between males and females.

**Extended Data Fig. 3:**
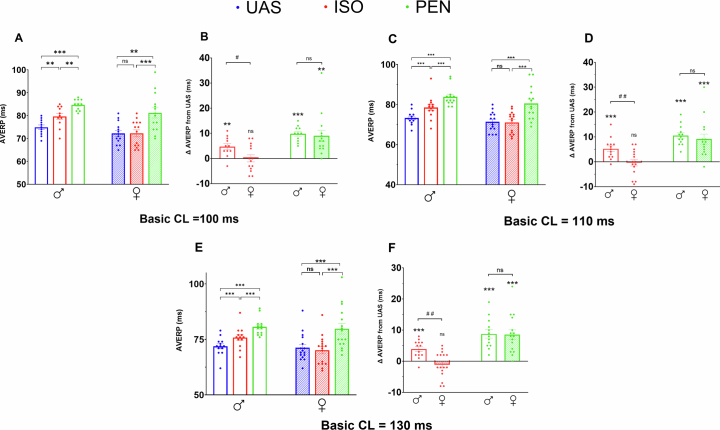
AVERP analysis for various basic CLs. **A:** Comparison of AVERP obtained with 100-ms basic CL under UAS, ISO and PEN, stratified by sex. **B:** Comparison between males and females. The Δ change in AVERP obtained with 100-ms basic CL relative to UAS under ISO and PEN conditions. **C-D:** Similar representations as in **A-B** but for 110-ms basic CL. **E-F:** Similar representations as in **A-B** but for 130-ms basic CL. Similar data for 120-ms basic CL are presented in Fig. 4C, D in the main paper.

**Extended Data Fig. 4:**
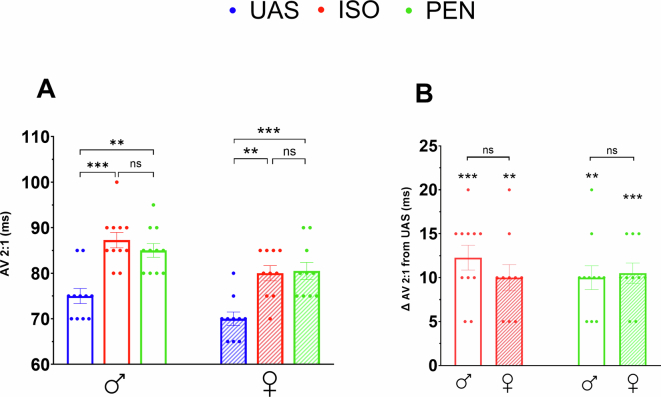
AV 2:1 block is prolonged by both ISO and PEN in both sexes. **A:** Comparison of AV 2:1 block under UAS, ISO and PEN, stratified by sex. Note the prolongation by both ISO and PEN relative to UAS in both males and females**. B:** Comparison between males and females. The Δ change in AV 2:1 block relative to UAS under ISO and PEN conditions. Statistical analysis: **A:** Friedman’s and Dunn’s multiple comparisons. **B:** Mann–Whitney test.

We next analyzed the effects of ISO and PEN on AV nodal properties. First, AVERP was tested using four different basic CLs (100, 110, 120 and 130 ms). As expected, considering AV nodal physiology, we noted a gradual, rate-dependent increase in AVERP with decreasing CL. This finding was noted in both sexes and was highly significant (*P* < 0.001 for both sexes, two-way ANOVA; Fig. 4a,b). Interestingly, both anesthetics markedly increased AVERP in males, although PEN had a greater effect than ISO. However, only PEN affected AVERP in females. Detailed analyses of the AVERP findings for a CL of 120 ms CL and the other CLs are shown in Fig. 4c,d and Extended Data Fig. 3, respectively, further supporting these findings. In contrast with the marked sex-dependent effect of ISO on AVERP, the effects of both anesthetics on the dynamic properties of the AV node (AV Wenckebach block and AV 2:1 block) were noted for both agents and in both sexes. However, for ISO, they were more prominent and significant in males (*P* = 0.001 versus *P* = 0.02, and *P* < 0.001 versus *P* = 0.001, for AV Wenckebach and AV 2:1 in males versus females respectively, Friedman test with Dunn’s multiple comparisons in both comparisons; Fig. 4e,f and Extended Data Fig. 4).

### ISO and, to a lesser extent, PEN inhibit the AF substrate in male rats

**Fig. 5:**
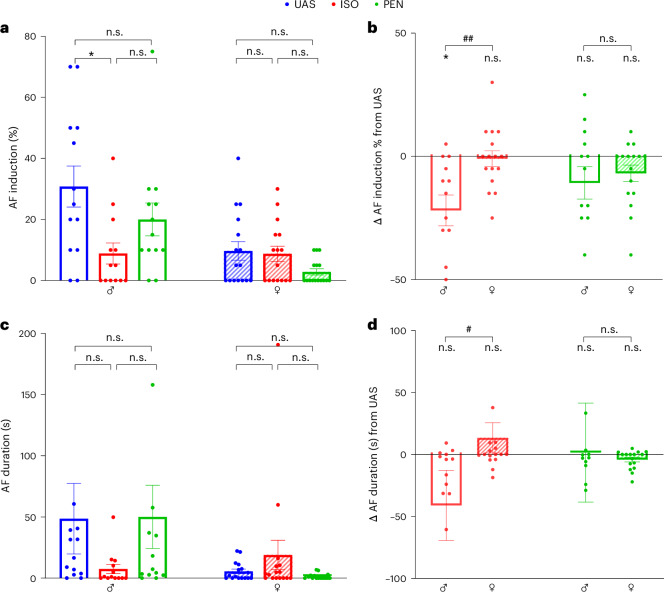
AF substrate is inhibited by ISO in males only. **a**, Comparison of AF induction (%) under UAS, ISO and PEN, stratified by sex. Note the significant inhibition by ISO relative to UAS in males only. **b**, Comparison between males and females of the Δ change in AF induction (%) relative to UAS under ISO and PEN conditions. Note the marked difference between the effect of ISO in males versus females. **c**, Comparison of AF duration under UAS, ISO and PEN, stratified by sex. Note the differential effect of ISO in males versus females for this parameter as well. **d**, Comparison between males and females of the Δ change in AF duration relative to UAS under ISO and PEN conditions. Statistical analysis: the comparison was performed using Friedman’s and Dunn’s multiple comparisons in **a** and **c**; the comparison was performed using the Mann–Whitney test in **b** and **d**. For clarity, two data points in **b**, two in **c** and five in **d** were out of scale and are not represented. In all panels, *n* = 13 for males and *n* = 16 for females. All data are expressed as mean ± s.e.m. *P < 0.05; ^#^*P* < 0.05, ^##^*P* < 0.01, for Δ change between males and females.

**Extended Data Fig. 5:**
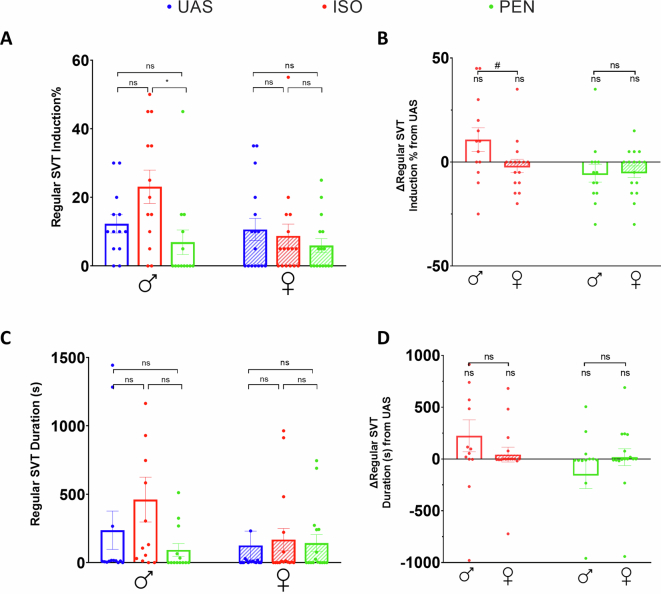
ISO increases the induction of regular supraventricular tachyarrhythmias in males only. **A:** Comparison of regular supraventricular tachyarrhythmias (SVT) induction (%) under UAS, ISO and PEN, stratified by sex. **B:** Comparison between males and females. The Δ change in SVT induction (%) relative to UAS under ISO and PEN conditions. **C-D:** Similar representations as in **A-B** but for SVT duration. Statistical analysis: **A, C:** Friedman’s and Dunn’s multiple comparisons. **B, D:** Mann–Whitney test. For clarity, two data points in **C** and two in **D** were out of scale and are not represented.

**Extended Data Fig. 6:**
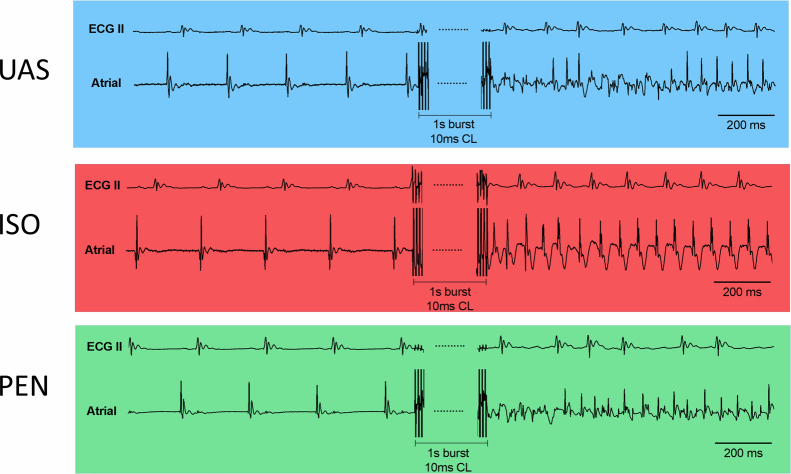
Typical recordings during the arrhythmia induction procedure. Recordings demonstrating induced atrial arrhythmias in the same male rat under UAS, ISO and PEN. Left: sinus rhythm ECG and atrial bipolar recordings at baseline. Middle: burst pacing protocol (1-second duration, 100 Hz, double the stimulation threshold). Right: post-burst responses. Note clear AF in UAS and under PEN, and regular SVT under ISO.

Finally, we comprehensively assessed the effects of the anesthetics on atrial arrhythmias induced by burst pacing in our rat model. Manual analysis of irregular (AF) substrate parameters revealed that ISO significantly decreased the induction of AF (%) in the males compared to UAS (*P* = 0.02, Friedman test with Dunn’s multiple comparisons), but there was no notable effect in the females (Fig. 5a). Comparison of the Δ changes in AF induction relative to UAS further stressed the inhibitory effect of ISO on the AF induction, which differentially affected males only (Fig. 5b). A similar inhibitory tendency of ISO was also noted regarding AF duration (Fig. 5c). However, using conservative statistical analysis, as is required for non-Gaussian distributions, this tendency was not significant. Comparison of the Δ changes in AF duration relative to UAS reached significance and again supported an inhibitory effect of ISO in males only (Fig. 5d). Interestingly, the inhibitory effect of ISO on the AF substrate parameters of males was associated with a tendency for increase in the induction of regular atrial arrhythmias, although this was not significant (Extended Data Figs. 5 and 6).

In contrast to ISO, the manual analysis of PEN’s effect on the AF substrate did not reveal a significant impact on AF induction or duration. However, a tendency for reduced induction was observed (Fig. 5a,b), which was further supported by the objective analysis described below.

**Fig. 6:**
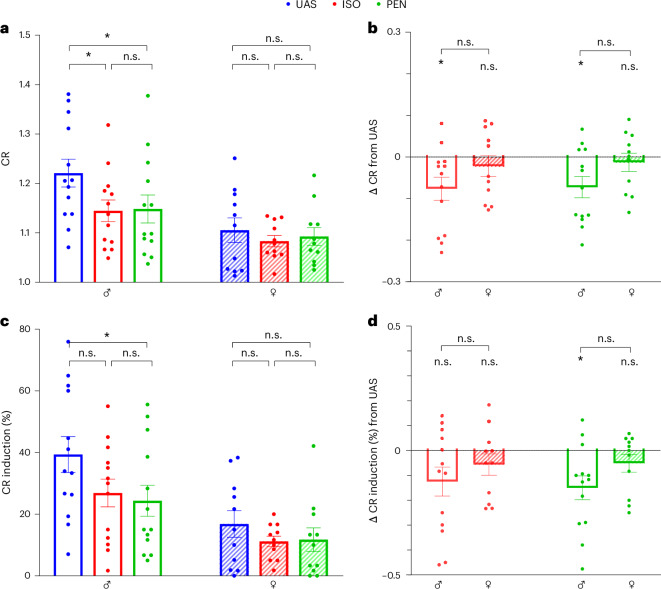
Objective AF substrate analysis indicates that both ISO and PEN inhibit AF substrate in males. **a**, Comparison of the mean CR of the first 5-s post-burst pacing under UAS, ISO and PEN, stratified by sex. Note the significant inhibition by both ISO and PEN relative to UAS in males only. **b**, Comparison between males and females of the Δ change in CR relative to UAS under ISO and PEN conditions. The results were nonsignificant in this analysis. **c**, Comparison of the percentage of signals above the CR threshold for irregular arrhythmias under UAS, ISO and PEN, stratified by sex. Inhibition of this parameter was noted only for PEN in the males. A nonsignificant inhibitory tendency was also noted for ISO in the males. **d**, Comparison between males and females of the Δ change in the percentage of signals above the CR threshold for irregular arrhythmias relative to UAS under ISO and PEN conditions. Statistical analysis: Friedman’s and Dunn’s multiple comparisons in **a** and **c**; Mann–Whitney test in **b** and **d**. In all panels, *n* = 13 for males and *n* = 16 for females. All data are expressed as mean ± s.e.m. *P < 0.05.

To further substantiate the above findings, we also analyzed the AF substrate of the rats using our recently developed objective tool, which aims to clean up the atrial signal from ventricular mixing, followed by calculation of the CR^38^ (see the Methods for a detailed description). As we described previously, the CR parameter detects irregular atrial signals in a highly accurate manner. A clear inhibitory effect of both anesthetics on the mean CR post-burst pacing was noted in males (Fig. 6a,b), but there was no notable effect in females. A similar tendency was noted when we analyzed the percentage of seconds above the arrhythmic CR threshold (Fig. 6c,d). However, these findings were only significant for PEN in males (*P* = 0.02, Friedman test with Dunn’s multiple comparisons).

**Fig. 7:**
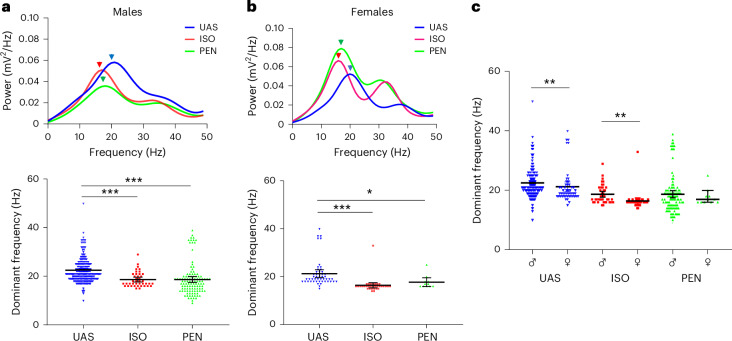
A lower AF dominant frequency is present under both anesthetics in both sexes. **a**, Top, average power spectrum of the AF signal in males under UAS, ISO or PEN. The arrowheads indicate the dominant frequency in each condition. Bottom, detected dominant frequency of all AF signals in each condition. **b**, Similar representation as in **a** but for females. Note that in both sexes there was a significantly reduced AF dominant frequency under ISO and PEN. **c**, Comparison of AF dominant frequency between males and females under each condition. Note the decreased dominant frequency in females compared with males under UAS and ISO. Statistical analysis: one-way ANOVA was applied, followed by post-hoc Tukey’s multiple-comparison test in **a** and **b**; the Student’s *t*-test was applied to compare males and females in **c**. In males, *n* = 193, 41 and 113 s windows for UAS, ISO and PEN, respectively. In females, *n* = 50, 33 and 11 s windows for UAS, ISO and PEN, respectively. All data are expressed as mean ± s.e.m. *P < 0.05, **P < 0.01, ***P < 0.001.

Finally, we performed a power spectrum analysis of the burst-pacing-induced AF signal under each condition. In both males and females, we found that each anesthetic significantly reduced the dominant frequency of the AF (*P* < 0.0001, *P* < 0.0001 in males, *P* < 0.0001, *P* = 0.014 in females, for ISO and PEN, respectively, Kruskal–Wallis test with Dunn’s multiple comparisons; Fig. 7a,b), a finding that may be consistent with the prolonged AERP seen under each anesthetic. In addition, compared with males, a lower dominant frequency was noted for females under UAS and ISO (Fig. 7c). Overall, this analysis may suggest that differences in the atrial electrophysiological properties of males versus females and in response to ISO and PEN not only modulate the AF substrate but also affect the characteristics of induced AF episodes. However, the contribution of extrinsic factors, such as autonomic activation of these properties, cannot be excluded (Discussion).

## Discussion

The current study is based on our recent advances in developing an implantable electrophysiological device adapted for atrial programmed stimulation protocols and AF substrate assessment in freely moving rats^35–38^. Using this device, we aimed to comprehensively characterize how ISO and PEN, which are routinely used in conventional, invasive electrophysiological studies, affect the electrophysiological results relative to the setting nearest to the physiological state, that is, the UAS. We characterized the results in both males and females to obtain a comprehensive overview of the effects of the selected anesthetics on the supraventricular electrophysiology. Our main findings indicate that, although conventional doses of ISO and PEN do not have a major role in modulating heart rate, they can affect multiple other aspects of rat supraventricular electrophysiology in a complex manner that often varies between the two agents and also varies between males and females. In general terms, our findings clearly stress the importance of considering any effects of the anesthetic agent used when electrophysiological results are reported. Indeed, while the use of anesthetic agents is inevitable in conventional rodent electrophysiology studies, there is great variability between studies in terms of the agents and doses used. Moreover, in many cases, the agent and/or the dose are not even mentioned in the relevant methods section (for example, refs. ^21,39^). Our current findings stress the need to change this problematic practice and further develop and use technologies that enable electrophysiological studies in the UAS. We discuss further specific findings and their implications below.

### Sex-dependent differences in the UAS supraventricular electrophysiology

Our initial comparison indicated important differences between the electrophysiological findings in males and females in the UAS (Table 1). These included slower heart rate, longer PR interval, prolonged AV Wenckebach and AV 2:1 blocks in males, and longer AERP and substantially reduced AF substrate in females. An important issue that should be considered is the increased weight of the males during the electrophysiology study. As our recordings were made 4 weeks after device implantation, this difference simply reflects the different growth rates between males and females. While we cannot discount that this weight difference may have affected the electrophysiological findings to some extent, at least for heart rate, such differences have also been reported previously from a study that carried out telemetric recordings in male and female rats of approximately similar weight^40^. Interestingly, in that study, the difference in heart rate was most pronounced between males and females that were housed singly^40^; this condition existed in our case as well, to prevent cage mates from extracting the electrophysiology devices. The prolonged AV Wenckebach block and AV 2:1 block, as well as the shorter AERP in males, might reflect either different intrinsic atrial properties or differences in extrinsic factors, such as autonomic tone or sex hormones. Further experiments, which were beyond the scope of the current study, will be needed to differentiate between such possibilities.

For the sex-dependent difference in AERP, the interpretation is somewhat more complex. We have previously shown that, in male rats implanted with an atrial electrode, AERP becomes progressively shorter over time^37^, presumably reflecting a remodeling process as a result of mechanical loading of the electrode on the right atrial myocardium. Thus, one possibility is that the atrium in females is less susceptible to the mechanical loading induced by the electrode, and therefore, the sex difference in AERP that was detected 4 weeks after device implantation mainly reflects less loading-dependent electrical remodeling in females. Indeed, it is well documented that estrogens improve myocardial adaptation to ventricular pressure overload in women who have hypertension^41^, and similar findings have been reported for rodent models of trans-aortic constriction^42,43^. This possibility may be further supported by the reported absence of sex-dependent differences in the action potential characteristics of mice atrial cardiomyocytes under basal conditions^31^. Our findings suggest that it may be useful in future work to perform a direct comparison of AERP in males and females shortly after implantation of the electrophysiology device. Such an experiment, although technically relatively simple to perform, was beyond the scope of our current study. As mentioned above, there were also remarkable sex-dependent differences in AF substrate. This issue will be explored in greater detail later in the discussion.

### ISO and PEN markedly modulate supraventricular electrophysiology

Methodologically, for each parameter, we compared the differences between UAS, ISO and PEN within each sex, and also performed a comparison of the Δ change from UAS in males and females. Initial analysis of the RR interval indicated that ISO did not affect the heart rate, while PEN had a modest bradycardic effect in both sexes. We could find no studies reported in the literature that repeatedly measured the heart rate of the same rodents under UAS as well as under ISO or PEN, as we did. However, some reported studies that used a different approach also indicated relatively minor effects of these agents on the heart rate of rodents^32,44,45^. While these findings may imply that the supraventricular electrophysiological properties retain their physiological values when under the influence of these two agents, our further analyses indicated that this was clearly not the case. Indeed, our analysis of CSNRT in the males revealed marked prolongation under ISO compared with under UAS or PEN. This finding may suggest that, while the pacemaker properties within the SA node remain unaffected in the presence of ISO (resulting in an unaltered heart rate), SA conduction pathways that transfer the excitation from the SA node to the atria^46,47^ are affected by ISO, leading to SA exit block and prolonged CSNRT. Moreover, because the SA conduction pathways share properties with the AV nodal conduction system^47^, the marked effects of ISO on AV nodal function may also support this hypothesis (Fig. 4). Interestingly, in both cases, the effects of ISO in males were far more prominent than in females, a finding that may deserve further attention in future studies to gain a mechanistic understanding.

Our findings indicated that AERP was markedly prolonged by both ISO and PEN. Interestingly, at least for ISO, similar findings have also been observed in humans^48,49^. However, it is difficult to conclude whether similar mechanisms are responsible for these observations in both rodents and humans. As AERP is a surrogate of atrial action potential duration (APD), we sought to compare our findings with reports describing the direct effects of ISO on cardiac APD. However, the findings of these reports are inconsistent and do not include data regarding atrial tissue or myocytes. A study involving isolated ventricular myocytes from guinea pigs demonstrated that the effects of ISO are complex and dose dependent, leading to prolonged APD at low concentrations (<2%) followed by a marked reduction in APD at higher doses^50^. The main mechanism leading to APD prolongation was attributed to the inhibitory effects of ISO on IK_dr_, a current that is not involved in action potential repolarization in rodents. Meanwhile, a study involving isolated rat ventricular cardiomyocytes suggested a reduction in APD due to marked inhibition of L-type Ca^2+^ current, with only a modest inhibitory effect on I_to_, the dominant repolarizing current in rodents^51^. Interestingly, it has also been found that desflurane, another volatile anesthetic with similarities to ISO, prolonged APD in isolated rat ventricular myocytes by markedly suppressing I_to_(ref. ^52^). It is possible that some of the above inconsistencies may be related to differences in doses and experimental conditions. In vivo, indirect effects through autonomic modulation may also play a role. In any case, our data support the notion that prolonged atrial APD is the dominant effect of the conventional concentration of ISO (that is, approximately 2%), at least in rats. Interestingly, while PEN prolonged AERP in both sexes in a similar manner, ISO had a greater effect in males, as also described above in relation to CSNRT and AV nodal properties. Regarding AERP rate dependence, our group has previously shown that this property is practically absent in male rats and mice under ISO anesthesia^32^, as well as in freely moving male rats^37^. Our current data confirm rather flat AERP rate dependence as a uniform finding under all conditions, although in females modest but significant reverse adaptation was noted. Importantly, our group has previously documented similar insensitivity of ventricular ERP to the pacing CL in UAS rats and mice^34,53^. Thus, at least in vivo, the absence of ERP rate dependence seems to be a consistent finding in the rodent myocardium.

### Modulation of AF substrate and AF signal by sex and anesthetics

Our electrophysiological studies were performed 4 weeks after electrophysiology device implantation. Thus, we had the opportunity to compare the effects of anesthetics on the AF substrate that progressively develops in our model over time^36–38^. Our first finding was that UAS females had markedly less AF substrate compared with males (Table 1). In addition, their AF substrate was somewhat insensitive to both anesthetics (Figs. 5 and 6). Several factors can contribute to the reduced AF substrate in females, including reduced atrial size and prolonged AERP. A recent study noted similar sex-dependent AF substrate differences in CD-1 mice under 2% ISO, which was mainly attributed to testosterone-dependent changes in connexin lateralization^31^. In this regard, it would have been helpful to determine the atrial conduction velocity in our rats. However, while our electrode could theoretically enable such recording^37^, we found that this was only practically possible in a minority of cases. In any case, the reduced AF substrate in females has important practical implications for the design of future AF studies. It will also be important to investigate if orchiectomy and ovariectomy affect the results, as was performed in mice^31^.

The inhibitory effect of ISO on the conventional AF substrate parameters of males is a finding of particular importance, considering the broad use of this agent in AF-related studies involving rodents. This finding was confirmed both by conventional measurements (AF induction and duration) and by our recently developed objective tool for the analysis of CR that dominantly detects irregular arrhythmias^38^. Using the latter approach, we also detected a reduction in AF substrate under PEN (Fig. 6), which we were unable to detect manually. An important consideration here is the signal resolution of the arrhythmic recordings obtained and the ability to differentiate between regular and irregular signals. Most conventional recordings, certainly those that use peripheral ECG to identify arrhythmias, cannot discriminate between totally regular SVTs and AF. Our findings highlight the importance of this issue by showing how ISO, which markedly increases AERP, leads to markedly reduced AF substrate concomitant with a clear tendency toward more regular SVTs (Extended Data Fig. 5). These changes could not have been identified using conventional low-resolution recordings and an analysis that pools regular and irregular arrhythmias. Thus, our findings stress the importance of obtaining high-resolution atrial signals for accurate AF substrate analysis. Of note, while there are no data in the literature that clearly elucidate the electrophysiological mechanisms underlying regular and irregular supraventricular arrhythmias in rats, our observations suggest that the former are far more stable and are, therefore, consistent with a single reentry cycle. Importantly, regular SVTs, such as AV nodal reentry tachycardia, may not even be dependent on the atrial tissue properties per se. Finally, the power spectrum analyses (Fig. 7) were also directly related to our current ability to record high-resolution atrial signals and digitally clean the signal from ventricular mixing^38^. Our analyses indicated that, in both males and females, ISO and PEN reduced the dominant frequency of the AF signals. It is tempting to speculate that these findings may be related to the AERP-prolonging effects of both anesthetics leading to increased AF wavelengths. Similarly, the lower dominant frequency in females under UAS and ISO (Fig. 7) may also be correlated with the increased AERP in females. However, at this stage it is difficult to know whether the differences in AERP were indeed the main determinant of these findings. Thus, further mechanistic studies should be performed to address these questions.

At this stage, it is difficult to determine whether our current findings in rats can be directly applied to other species. Given the similarities in cardiac electrophysiology between rats and mice, it is plausible that our data may also be relevant to mice. However, further studies using methodologies similar to those used here are needed to address this question more directly. Regarding large mammals and humans, we did not find detailed information on the effects of PEN. However, as mentioned earlier, observations in humans under ISO are highly reminiscent of our findings, particularly in terms of AERP and AVERP prolongation^48,49^. In addition, studies in dogs have reported AERP and AVERP prolongation, as well as an anti-AF effect of ISO^54,55^. These findings suggest that the underlying mechanisms leading to the observed effects of ISO may not be limited to rats. Nevertheless, we cannot rule out the possibility that the apparently similar phenotypes observed in rats and large mammals result from different underlying mechanisms producing similar outcomes. Further studies are needed to address this issue more conclusively.

## Methods

### Animals

This study was conducted in strict accordance with the Guide for the Care and Use of Laboratory Animals of the National Institute of Health. All animal studies reported in this study were approved by the institutional ethics committee of the Ben-Gurion University of the Negev, Israel (protocol no. IL42062021D). Adult male and female Sprague–Dawley rats were obtained from Harlan Laboratories. Experiments were performed on male and female rats with a body weight of approximately 250 g at the time of surgery (~6 weeks old for males and ~8 weeks old for females, respectively). The animals were kept under standardized conditions throughout the study, according to home office guidelines: 12:12-h light:dark cycles at 20–24 °C and 30–70% relative humidity. Animals had free access to autoclaved rodent chow and to water purified by reverse osmosis. The animals were monitored on a daily basis for signs of stress or unusual weight loss, according to guidance from Ben-Gurion University veterinary services (assured by the Office of Laboratory Animal Welfare, USA #A5060-01 and fully accredited by the Association for Assessment and Accreditation of Laboratory Animal Care International). After all electrophysiological evaluations, the rats were euthanized under deep anesthesia.

### Electrophysiology device and surgical implantation procedure

The electrophysiology device used in this study and details about the atrial quadripolar electrode have been described in detail elsewhere^36,37^. In brief, the device comprises an eight-pin connector that is attached by highly flexible, insulated electrical wires (AS155-36, Cooner Wire) to the atrial quadripolar electrode as well as to three peripheral ECG leads. The atrial quadripolar electrode contains four platinum–iridium electrical poles that are embedded in medical-grade silicon (MED-6219P) and fixed to the tissue by miniature stainless-steel hooking pins (26002-10, Fine Science Tools). For implantation, the animals were anesthetized with ketamine/xylazine (intramuscular, 75/5 mg/kg). Rats were mechanically ventilated and placed on a heating pad (37 °C). Upper-right thoracotomy was performed under sterile conditions, and the atrial pericardium was removed. The atrial quadripolar electrode was implanted on the epicardial surface of the right atrium, and the ECG electrodes were subcutaneously positioned in the left forelimb, right forelimb and left leg. After the chest was closed, the eight-pin connector was exteriorized through the skin on the rat’s back, and a shielding ring with four plastic restraints was used to prevent the device from being extracted over time. The ring was inserted over the connector, sutured to the skin and glued to the connector over the four plastic restraints^36^. This minimized the risk of the connector being extracted by other rats. After conventional postoperative recovery, the animals were maintained in normal cages for 4 weeks to allow sufficient AF substrate to develop^36,37^. Thereafter, repeated electrophysiological measurements were conducted, as described below.

### Experimental design of the repeated electrophysiological evaluations

Thirty days after device implantation, the animals underwent three consecutive electrophysiological measurements (Fig. 1). For the initial UAS electrophysiological evaluation, each animal was placed in a dedicated electrophysiology cage. The back connector on each rat was attached by an elastic cable to the pacing and recording apparatus via a multichannel commutator (PLA-SL12C/SB, Plastics One), allowing the rat to move freely in its cage without affecting the electrical connections. In each animal, a pair of atrial poles was selected for pacing and electrically connected to an optically isolated pacing unit (STG4002-16 mA, Multichannels). The remaining two atrial poles and the three peripheral ECG electrodes were connected to a voltage amplifier (Amplifier 1700, A-M Systems). As previously described, the electrode side that was used for pacing was empirically determined on the basis of a relatively low capture threshold and the ability to differentiate the atrial signal from the stimulus artifact in the recordings from the other side. Once a pacing and recording configuration had been selected, it remained without changes throughout the repeated electrophysiology studies conducted under anesthesia. Signals were filtered (1–1,000 Hz with a notch filter at 50 Hz) and sampled to a PC at a digital sample rate of 2 kHz. A self-made program developed using LabVIEW 7.1 (National Instruments) controlled data acquisition and electrical stimulation. UAS electrophysiology studies were performed in freely moving rats, after overnight adaptation to the electrophysiology cages and during daylight hours, that is, the part of the circadian cycle when animals were inactive, as previously described^36,37^. After the UAS electrophysiology procedure, each animal was moved to its regular cage for 24 h. Subsequently, a second electrophysiology study was performed under ISO anesthesia (2% in O_2_ mixture) in a manner identical to the first one. For the procedures carried out in anesthetized rats, a rectal temperature probe was inserted, the animals were placed on a heating pad and the temperature was maintained at approximately 37.5 °C throughout the electrophysiology study. Of note, while we could have performed the ISO electrophysiology study under lower levels of ISO (as low as 1%), we intentionally selected 2% as this is the most commonly used level of ISO in acute electrophysiology studies that use conventional invasive catheters^20^. Lastly, following the ISO electrophysiology procedure, each animal was moved to its regular cage for a recovery period of 72 h, and then the final electrophysiology study was performed under PEN anesthesia (intraperitoneal, 40 mg/kg). At the end of this study, the experiment was terminated, and the rats were euthanized with a high dose of PEN.

The electrophysiological evaluation was performed as previously described^37^. RR and PR intervals were obtained using the average of five consecutive cycles on the nonpaced ECG. Following baseline recordings, the atrial pacing threshold was obtained using bipolar square current pulses (total duration 4 ms, 2 ms in each direction), and the stimulus intensity was raised to double threshold for the remainder of the electrophysiology study. A programmed S1–S2 stimulation protocol (S1 = 10) was used to determine AVERP and AERP, in the millisecond range. To assess rate dependence, the S1–S1 cycle (basic CL) was varied between 130 ms and 100 ms for the AVERP measurements and 120–70 ms for the AERP measurements. All AVERP and AERP values were confirmed three consecutive times. SA node recovery time (SNRT) was evaluated using burst-pacing protocols (30 s, 120 ms basic CL) applied three times with a pause of 30 s between bursts. Spontaneous CL was measured after each SNRT burst, calculated as the average of three consecutive spontaneous beats, based on our previously reported SNRT calibration pilot^37^.

### Arrhythmic substrate analysis

The arrhythmic substrate evaluation in each of the three electrophysiology studies (UAS, ISO and PEN) comprised 20 consecutive triggering bursts (1 s duration, 10 ms CL). Arrhythmic episodes lasting more than 4 min were aborted using short (1 s) pacing bursts of increasing intensity until sinus rhythm was restored. The minimum time between pacing bursts was 1 min from the end of an event. If an episode of >60 s was detected, the delay from the end of this episode to the next pacing burst was equal to the duration of the AF episode. The cutoff for defining a positive arrhythmic event was defined as >1 s following the burst-pacing protocol. To avoid any bias in the AF analysis caused by regular stable arrhythmic episodes, we distinguished between regular and irregular events in our analysis. As we recently reported^56^, we have previously noted that regular arrhythmic episodes are characterized by a stable CL of >60 ms. Thus, in the current study, we defined AF as rapid irregular atrial ECGs or atrial waveforms in which the main repeating component had a duration of <55 ms. Regular arrhythmic waveforms were analyzed separately from the AF analysis.

In addition to the manual analysis described above, we applied our recently developed computerized algorithm to clean any ventricular mixing from the atrial signal and thereafter quantity AF substrate and complexity in an objective manner, as described in detail previously^38^. In brief, pre-burst ventricular-complex sampling was initially performed, followed by its automatic subtraction from the entire atrial signal based on QRS detection in the ECG wave form. Next, the pure post-burst atrial signal was divided into 1 s windows, and the complexity of each window was analyzed by applying the Lempel–Ziv complexity algorithm^38^. The final CR value was calculated for each window by normalizing its Lempel–Ziv value to the pre-burst value in the same trace (Extended Data Fig. 1). CR values close to 1 indicate low complexity that is similar to the pre-burst sinus rhythm values. By contrast, values greater than 1 indicate increased episode irregularity relative to the pre-burst signal. Our detailed analysis previously revealed that a cutoff value of CR of 1.236 is ideal for accurately distinguishing regular rhythms from irregular events (AF)^38^. Finally, for the power spectrum analysis of the AF waveforms, conventional fast Fourier transform was performed, as previously described^38,56^. The recordings were all performed in the presence of a notch filter to reduce the signal levels proximal to 50 Hz, so an artificial decrease was detected around this frequency.

### Statistical analysis

A total of 29 rats (13 males and 16 females), in which the electrophysiology device was successfully implanted and demonstrated suitable atrial pacing and recordings 4 weeks after implantation, were included in our final analysis. Data analysis was performed using Prism 9.0 (GraphPad Software). All data are expressed as mean ± s.e.m. We used the Shapiro–Wilk test to examine whether various parameters were normally distributed. For parameters with a normal distribution in all groups, we used one-way ANOVA for repeated measurements, followed by post-hoc Tukey’s multiple-comparison test. For parameters that lacked a normal distribution, Friedman’s test was used with post hoc Dunn’s multiple-comparison correction. For direct comparisons between males and females in the UAS (Table 1), we used the Student’s *t*-test and the Mann–Whitney test for parameters that passed or failed the normality test, respectively. Comparisons of AERP and AVERP under different basic CLs were performed using two-way ANOVA for repeated measurements. AF substrate parameters generally do not exhibit a normal distribution, so we analyzed them all using nonparametric tests. Comparisons of delta values from the UAS with those with each anesthetic drug (ISO or PEN) between males and females were conducted using the Mann–Whitney test. AF dominant frequency was analyzed using the Kruskal–Wallis test with Dunn’s multiple comparisons. For all tests, the significance threshold was set at a two-sided *P* value <0.05. The specific tests that were used are mentioned in the legends of each figure and table.

### Reporting summary

Further information on research design is available in the Nature Portfolio Reporting Summary linked to this article.

## Online content

Any methods, additional references, Nature Portfolio reporting summaries, source data, extended data, supplementary information, acknowledgements, peer review information; details of author contributions and competing interests; and statements of data and code availability are available at 10.1038/s41684-025-01532-5.

## Supplementary information

Reporting Summary

## Data Availability

The datasets generated during and/or analyzed as part of this study are available from the corresponding author upon reasonable request.
